# Novel *KCNH1* Mutations Associated with Epilepsy: Broadening the Phenotypic Spectrum of *KCNH1*-Associated Diseases

**DOI:** 10.3390/genes12020132

**Published:** 2021-01-21

**Authors:** Randi von Wrede, Monika Jeub, Idil Ariöz, Christian E. Elger, Hubertus von Voss, Hanns-Georg Klein, Albert J. Becker, Susanne Schoch, Rainer Surges, Wolfram S. Kunz

**Affiliations:** 1 Department of Epileptology, University Hospital Bonn, 53127 Bonn, Germany; randi.von_wrede@ukbonn.de (R.v.W.); susanne.schoch@uni-bonn.de (S.S.); rainer.surges@ukbonn.de (R.S.); 2 Beta Neurologie GmbH, 53227 Bonn, Germany; monika.jeub@betaklinik.de (M.J.); christian.elger@betaklinik.de (C.E.E.); 3 Section for Translational Epilepsy Research, Department of Neuropathology, University Hospital Bonn, 53127 Bonn, Germany; idil.uyan@gmail.com (I.A.); albert_becker@uni-bonn.de (A.J.B.); 4 Center for Human Genetics and Laboratory Diagnostics, Dr. Klein, Dr. Rost and Colleagues, 82152 Martinsried, Germany; hubertus.vonvoss@medizinische-genetik.de (H.v.V.); hann-georg.klein@medizinische-genetik.de (H.-G.K.); 5 Department of Experimental Epileptology and Cognition Research, University Hospital Bonn, 53127 Bonn, Germany

**Keywords:** epilepsy, *KCNH1* mutations, Kv10.1 potassium channel

## Abstract

Here, we describe four patients suffering from a rather broad spectrum of epilepsy-related disorders, ranging from developmental and epileptic encephalopathy with intellectual disability (DEE) to genetic generalized epilepsy (GGE), which all harbor novel *KCNH1* mutations. In one family, we found a weak association of a novel nonsense mutation with epilepsy, suggesting reduced penetrance, and which shows, in agreement with previous findings, that gain-of-function effects rather than haploinsufficiency are important for the pathogenicity of mutations. De novo missense variants in the pore region of the channel result in severe phenotypes presenting usually with DEE with various malformations. The potential pathogenicity of a novel *KCNH1* germline mutation located outside of the critical pore domain observed in a GGE patient with a milder phenotype is supported by the fact that the very same amino acid exchange was detected as a somatic mutation in the resected brain tissue of a patient suffering from a focal cortical dysplasia type IIb. Thus, our case series broadens the phenotypic spectrum of *KCNH1*-associated diseases.

## 1. Introduction

Heterozygous missense mutations of *KCNH1* (Kv10.1) have been reported to cause the rare developmental disorders Zimmermann–Laband syndrome (ZLS) and Temple–Baraitser syndrome (TMBTS). ZLS is characterized by intellectual disability, epilepsy, facial dysmorphism, gingival enlargement, hypertrichosis and hypoplasia/aplasia of nails and terminal phalanges [1], whereas TMBTS patients typically display intellectual disability, epilepsy and broad thumbs and great toes with absent/hypoplastic nails [2]. More recently, pathogenic *KCNH1* mutations have been identified in uncharacterized patients with intellectual disability, epilepsy and variable skeletal abnormalities, which could be ascribed neither to a ZLS nor to a TMBTS phenotype [3]. Furthermore, a *KCNH1* mutation originally described in a ZLS patient was also found in a patient with a TMBTS phenotype [4]. This phenotypic variability and the great overlap between both syndromes suggest that ZLS and TMBTS are a continuum of the same disease. In this report, we present patients harboring novel missense mutations in the *KCNH1* gene suffering from epilepsy, but otherwise presenting distinct clinical features, broadening the phenotypic spectrum of *KCNH1* mutations. The missing strong association of a novel nonsense mutation with epilepsy in one family indicates that gain-of-function effects, rather than haploinsufficiency, are important for pathogenicity.

## 2. Materials and Methods

### 2.1. Human Subjects and DNA Collection from the Paraffin Samples

Genomic DNA from blood was isolated by routine techniques [5]. After surgical procedure, the fresh resected tissue was stored at −80 °C and embedded in paraffin. Hematoxylin–eosin (H&E) staining was performed to confirm the dysmorphic neurons and balloon cells characteristically observed in focal cortical dysplasia type IIb (FCD IIb). Dysplastic and the adjacent healthy tissue was defined, marked and the percentage of dysmorphic and balloon cells was are semiquantitatively estimated on the H&E staining by an experienced neuropathologist. Tissue from the dysplastic and healthy regions was collected separately by scratching the paraffin tissue from 10 consecutive slices of 10 µm thickness. Genomic DNA was isolated with the QIAamp^®^ DNA mini kit (Qiagen, Hilden, Germany) according to the protocol provided by the manufacturer.

### 2.2. Exome Sequencing

Whole exome sequencing (WES) was carried out as previously described [6], resulting in a 79-fold mean coverage (30-fold coverage for 88%, and 10-fold coverage for 97% of target sequences, respectively). Reads were mapped and variants were annotated as described. Filtering and variant prioritization were performed using the VARBANK database and analysis tool [7] at the Cologne Center for Genomics. In particular, we filtered for high-quality (coverage >15-fold, phred-scaled quality >25) and rare variants (MAF ≤ 0.001 based on dbSNP build 135; the 1000 Genomes database build 20110521; and the public Exome Variant Server, NHLBI Exome Sequencing Project, Seattle, build ESP6500) with predicted effects on protein sequence or splicing. To exclude pipeline-related artifacts (MAF ≤ 0.01), we filtered against variants from in-house WES datasets from 511 epilepsy patients. Verification of variants and testing of family members were performed by direct sequencing of purified PCR products performed by a commercial service.

### 2.3. Bioinformatical Analyses and 3D Structure Prediction

Multiple sequence alignment of the *KCNH1* gene in different organisms was performed using Clustal Omega. 3D structure prediction of human *KCNH1* wild type (Uniprot: O95259) and *KCNH1* p.Val713Glu (Uniprot entry: O95259) was performed by using Robetta [8], using the default parameters of the Tr-Rosetta software package. The stereochemical quality of the protein structure was assessed based on the Ramachandran plot of the residues using PROCHECK [9], and the model with the highest score was selected. For structure visualization and residue selection, a PDB file was uploaded onto the 3D Structure Viewer from the UCLA–DOE Molecular Biology Institute Server.

## 3. Results

### 3.1. Case Reports

#### 3.1.1. Case 1

A boy, aged 6 years and 4 months, was referred at the age of five years because of pharmacoresistant epilepsy of unknown origin at the time of admission. He was born by C-section after an uneventful pregnancy (36 gestational weeks) to healthy parents. At the age of seven months, a sagittal synostosis was surgically treated. He reached all milestones on time until the age of two. At the age of two and a half, a febrile seizure occurred. Since the age of three, he started to display generalized tonic–clonic seizures, generalized tonic seizures, myoclonic seizures, generalized nonmotor seizures, generalized atonic seizures with sudden falls and nonconvulsive status epileptici. Seizure frequency was high, with several seizures per day. Following nonconvulsive status, he was severely affected, with reduced responsiveness for several days. After the start of unprovoked seizures, his psychomotor development was severely impaired, and he lost already-achieved functions. An epileptic encephalopathy was diagnosed. Neuropsychological testing at the age of 5 years and 3 months revealed a global delay of childhood development, and he was rated at the level of a 20-month-old child. In clinical examination, he presented slight craniofacial dysmorphic signs with an oblique eyelid axis, hypertelorism and an open mouth, but no main characteristics of TMBTS/ZLS, such as nail hypoplasia of the great toes or thumbs. His muscle tone was reduced and he was ataxic, which, at least in part, might be an effect of antiseizure medication. Repeated MRIs were normal except for a mega cisterna magna and a temporal arachnoidal cyst on the left side. Simultaneous video-EEG monitoring revealed sporadic spike–wave and sharp–slow wave sequences. He had already received multiple antiseizure drugs (valproic acid, oxcarbazepine, levetiracetame, ethosuximide, lamotrigine, sultiame, phenobarbital, clobazam, topiramate, acetazolamide), a ketogenic diet and folic acid with no, or only, transient efficacy. At the age of 5 years and 5 months, he received a 5-day intravenous cortisone therapy to treat a nonconvulsive status, and perampanel was added to his topiramate monotherapy. Since then, generalized nonmotor seizures, generalized atonic seizures and nonconvulsive status epileptici have not been observed. As he still presented generalized tonic–clonic seizures several times a week, an additional therapy with dronabinol and vagal nerve stimulation therapy was initiated. Later on, perampanel and topiramate were phased out. Upon this, his attention and motor skills improved. The seizure situation remained stable. Trio-based whole exome sequencing revealed a de novo heterozygous variant in the Exon 6b of the *KCNH1* gene (NM_172362.2:c.596A>G, p.Lys199Arg). Preliminary clinical data for this patient have previously been reported [10].

#### 3.1.2. Case 2

**Figure 1:**
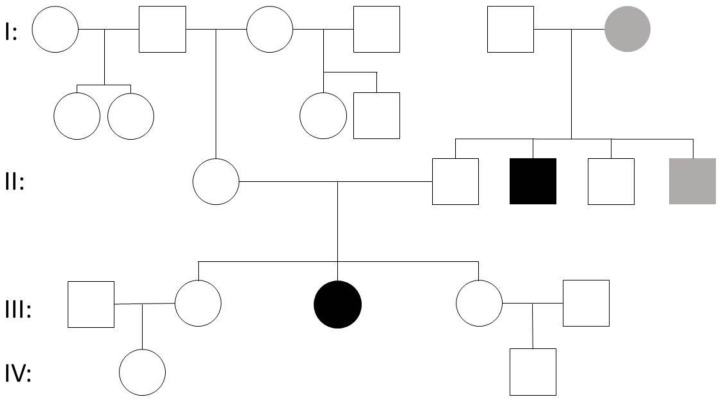
Pedigree of case 2. Please note that not only the index patient (III:3), but also her healthy father (II:2) and one healthy sister (III:2) harbor the *KCNH1* nonsense mutation p.Arg535*. Two brothers of the father (II:3 (intellectual disability) and II:5 (CNS tumor)) and the paternal grandmother (I:6 (CNS tumor)) of the index patient have a known history of CNS disorder.

This 36-year-old female was admitted for presurgical assessment of pharmacoresistant epilepsy of unknown origin at the time of admission. Seizures started at the age of two, with epileptic seizures characterized by an initial cry followed by involuntary contractions of the head, neck and trunk. Later on, she presented atonic seizures as well. EEG at that time showed multifocal hypersynchronous activity. Under a combination of sultiame, phenytoin and clonazepam, no more seizures were reported, therefore anticonvulsive medication was phased out at the age of six. At the age of sixteen, a generalized tonic–clonic seizure occurred. EEG at that time revealed a recurrence of pathological EEG activity with a sharp wave focus frontocentral on the left side, extensive sharp waves parieto-occipital on the right side, with a tendency of generalization and an extensive activation of hypersynchronous activity during sleep. Therapy with sultiame and clonazepam was reinstalled and no more seizures were reported for thirteen years. Focal onset seizures with impaired awareness started at the age of 14. Antiseizure medication with valproic acid, oxcarbazepine, lacosamide, lamotrigine and levetiracetam did not lead to seizure freedom. On admission, seizure frequency was one to three per week. Antiseizure medication consisted of lamotrigine and levetiracetam. Her medical history was normal. The family history displayed CNS disease in two brothers of the paternal line as well as the parental grandmother (Figure 1). Neurological, psychiatric and physical examination revealed no abnormalities. In particular, there were no skeletomuscular or cutaneous abnormalities. Neuropsychological testing showed an impairment of intelligence (HAWIE-R: IG = 67.35) with most prominent deficits in visuospatial performance and a reduced speed of perception consistent with a frontal impairment. Repeated MRIs and ECG were normal. Video-EEG monitoring for a duration of 48 h documented an α-background with intermittent theta activity in the left frontotemporal area, pronounced by hyperventilation, and sleep-activated sharp/sharp–slow wave activity in the left frontotemporal, bifrontotemporal and rarely in the right temporal areas. As results were consistent with genetic generalized epilepsy, whole genome exome sequencing was performed. A heterozygous nonsense mutation NM_172362.2:c.1603C>T; p.Arg535* in exon 8 of the *KCNH1* gene was detected. Analysis of parents and one sister revealed that the clinically unaffected father and sister carried both the same *KCNH1* nonsense mutation. Molecular genetic analysis of the other sister and the brothers of the father was not possible, as permission was not given.

#### 3.1.3. Case 3

This 40-year-old female patient was admitted to our tertiary epilepsy center due to pharmacoresistant epilepsy of unknown origin at the time of admission. Epileptic seizures started at the age of 13 and comprised daily absences, daily generalized atonic seizures and generalized tonic–clonic seizures once a year. Seizures had already led to relevant traumas such as fracture of the jaw and tooth loss. Carbamazepine, oxcarbazepine, valproic acid, topiramate, levetiracetam, pregabaline and zonisamide were previously administered without seizure control. On admission, the antiseizure medication consisted of lamotrigine, perampanel, primidone, clobazam and lacosamide. Except for recently started acoustic hallucinations, the neurological, psychiatric and physical examinations were normal. Despite high anticonvulsant medication, there were no signs of intoxication. In particular, there were no skeletomuscular or cutaneous abnormalities and her intelligence was normal. Her medical and family history were unremarkable. A cranial MRT and ECG revealed no abnormalities. The EEG showed an α-background with right and left temporal theta/delta slowing, as well as interictal right and left temporal sharp–slow wave activity and groups of generalized tonic spikes for 2–4 s during sleep. Video-EEG monitoring for a duration of 96 h documented four seizures with different origins and propagation consistent with genetic generalized epilepsy. To improve seizure control and under the idea of reduction of the drug load, the drugs lacosamide and clobazam were phased out, and primidone was switched to phenobarbital. Neither reduction nor increase in seizure frequency was apparent. Then, cannabidiol was started and increased stepwise to a daily dose of 330 mg. Upon this, a profound reduction in seizure frequency could be achieved, with atonic seizures occurring only twice in 3 months. Tolerance of the medication was good. The EEG pattern improved with an absence of epileptic discharges and long phases of normal activity. Whole exome sequencing revealed a variant in the *KCNH1* gene (NM_172362.2:c.2138T>A; p.Val713Glu), which was verified by Sanger sequencing. The mutation was predicted by different in silico tools (PolyPhen-2, MutationTaster, SIFT and PROVEAN) to be probably damaging/disease-causing. Analysis of first-degree relatives revealed that the clinically unaffected mother carried the same mutation, whereas the 48-year-old healthy sister displayed no *KCNH1* mutation. Molecular genetic analysis of the father was not possible, as he was already deceased.

#### 3.1.4. Case 4

A 40-year-old male patient suffered from structural focal epilepsy. Seizures started at the age of 5 years and became drug resistant. At the age of 27, epilepsy surgery (right prefrontal lesionectomy and multiple subpial transections) was carried out after noninvasive and invasive presurgical evaluation revealed right frontal epilepsy. After ten years of rare, less disabling seizures, he experienced seizure freedom for 13 years. Antiseizure medication was reduced, but not phased out due to an estimated higher risk of seizure recurrence considering pre- and postsurgical evaluation. One provoked seizure happened at the age of 40. The histopathology evaluation of the resected tissue confirmed an FCD IIb. Under treatment with a combination of phenytoin, phenobarbital and oxcarbazepin, he achieved again seizure freedom again. His family history was unremarkable. Exome sequencing identified in the resected FCD tissue a somatic c.2138 T>A (p.Val713Glu) mutation in *KCNH1*, which was virtually absent in the healthy brain tissue. No further somatic or germline mutations relevant for epilepsy were detected. Molecular genetic analysis of the parents was not possible, as they were already deceased.

### 3.2. De Novo Missense Mutations, but Also Germline Missense Mutations with Reduced Penetrance and Somatic Missense Mutations in KCNH1 Are Associated with Epilepsy

The above presentation of very different cases of patients with epilepsy shows that both the p.Lys199Arg de novo mutation but very likely also the p.Val713Glu germline missense mutation showing reduced penetrance appear to be strongly associated with epilepsy.

**Figure 2:**
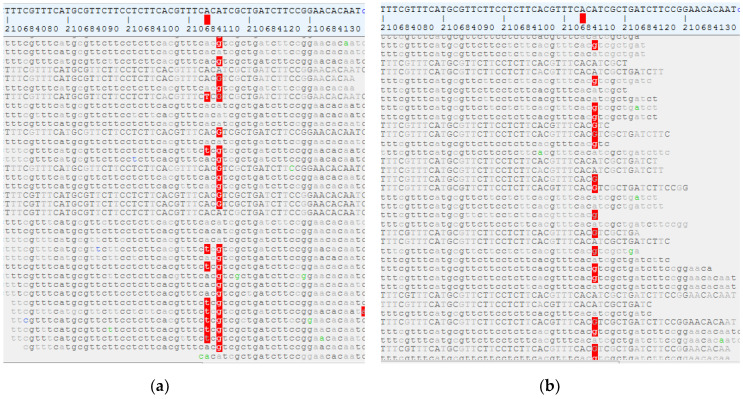
Somatic c.2138 T>A (p.Val713Glu) mutation in *KCNH1* in focal cortical dysplasia (FCD) tissue (**a**) detected by exome sequencing. Please note the virtual absence of the mutation at genomic position 1:210684113 A>T (position of the red cursor) in healthy brain tissue from the same patient (**b**) and in the blood sample (not shown).

**Figure 3:**
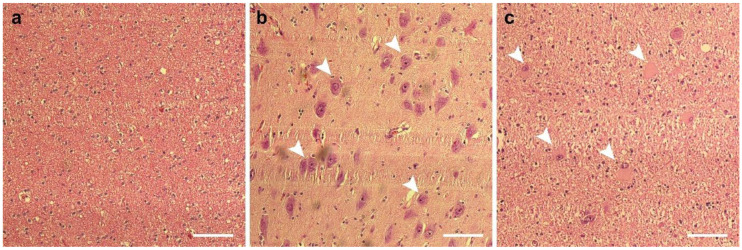
Hematoxylin–eosin staining of the surgically resected brain tissue from case 4. Adjacent healthy control tissue (**a**) from the same brain. Dysmorphic neurons (**b**) and balloon cells (**c**) from the lesion. Scale bars (**a**–**c**) 100 µm. Abnormal cells are semiquantitatively estimated to compose approximately 50% of the cell population in the collected lesion tissue (not shown).

The p.Val713Glu mutation detected as a germline variant in case 3 is located outside of the critical pore region close to the carboxy-terminus of the channel. The proof of its potential pathogenicity is related to the fact that the very same mutation was detected in the epileptogenic FCD IIb lesion from case 4 as a somatic mutation (Figure 2a), appearing under these circumstances also de novo. Whole exome sequencing of genomic DNA isolated from the lesion (Figure 2a) and the adjacent healthy tissue (Figure 2b) from the same patient revealed that this mutation was only observed in the lesion. H&E staining confirmed the presence of dysmorphic neurons and balloon cells in the resected tissue (Figure 3a–c)—the hallmarks of FCD IIb. Other potential epilepsy-related mutations were not detected in the WES analysis of the blood and healthy brain tissue samples from this patient (case 4). Moreover, MTOR pathway-related somatic mutations, also reported to be frequently detectable in FCD IIb lesions [11,12], were completely absent in the WES analysis of the resected lesional brain tissue from patient 4.

### 3.3. Evolutionary Conservation of the Mutated Residue and Computational Prediction of the 3D Structure of KCNH1 p.Val713Glu

**Figure 4:**
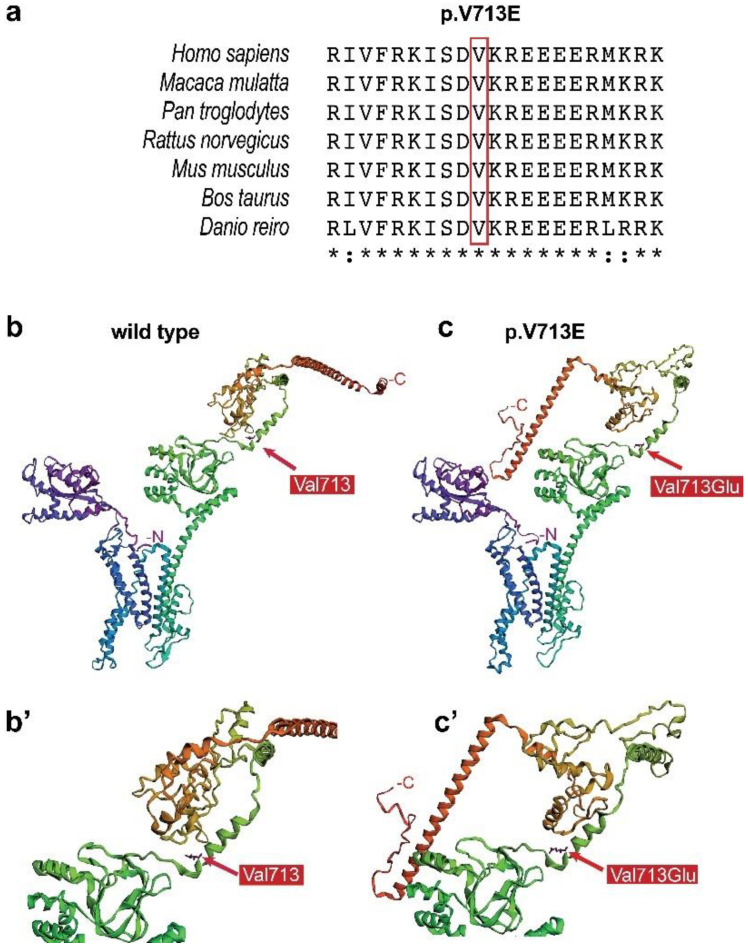
Conservation of the mutated valine among different species (**a**). 3D protein structure prediction of the wild type (**b**), and *KCNH1* V713E (**c**), and a zoom in into the mutated residue (**b’**,**c’**). The missense point mutation is predicted to induce a different folding pattern at the C-terminus.

The *KCNH1* gene codes for a potassium channel protein that is composed of 989 amino acids. Alignment of the protein sequence of *KCNH1* from seven different species showed that the valine residue at position 713 is highly evolutionary conserved (Figure 4a). In order to assess if the amino acid exchange from valine to glutamic acid impacts the 3D structural organization of *KCNH1*, and thereby potentially its function, we have performed a prediction of the 3D structure of the wild-type and the mutated protein (Figure 4b,c) using the Robetta algorithm. This analysis revealed that the exchange of valine against glutamic acid at position 713 resulted in a prominent structural rearrangement, whereby the C-terminal helical region changes orientation and orients itself towards the N-terminus.

## 4. Discussion and Conclusions

The actual impact of the identified novel mutation on the function of the *KCNH1* channel protein is not known yet. In previous studies, 10 pathogenic gain-of-function *KCNH1* variants have been identified as de novo mutations that lead to clinically well-defined syndromes such as Temple–Baraitser syndrome [2] and Zimmermann–Laband syndrome [1], which show dysmorphic features, intellectual disability and characteristics of an epileptic encephalopathy. Here, we present three novel pathogenic variants covering a very broad phenotypic spectrum (Table 1).

Our case 1 harboring the p.Lys199Arg de novo mutation fits in general to the classical disease spectrum [10], although typical dysmorphic signs of TMBTS/ZLS such as nail hypoplasia of the great toes or thumbs were absent. The mutation is located in front of the critical pore domain ranging from amino acid positions 218 to 509. This might be the reason for the absence of these typical dysmorphic signs reported for the classical TMBTS/ZLS phenotype.

It has been previously described that loss-of-function variants in the gene were better tolerated [1,2]. In agreement with this notion, our patient with the p.Arg535* mutation presented with genetic generalized epilepsy (GGE) combined with intellectual disability. However, the healthy father and one healthy sister of the patient were also identified as mutation carriers, pointing to a quite low penetrance of this particular nonsense variant. This is in line with the presence of eight nonsense variants in the large control cohort of about 125,000 individuals reported by GnomAD [13], indicating only moderate constraints of the *KCNH1* gene for nonsense variants.

We identified a further rare variant, p.Val713Glu, located at the C-terminus of the channel protein. The functional relevance of this alteration is supported by the fact that we detected this variant not only as a germline mutation in a GGE patient, but also as a somatic variant in the resected brain tissue of a patient suffering from focal cortical dysplasia type IIb. Under this circumstance, the p.Val713Glu mutation appeared obviously also de novo. In the absence of other somatic mutations, such as classical mTOR pathway mutations [11,12], which could contribute to the epileptogenicity of the FCD IIb lesion, it is reasonable to assume that this mutation is responsible for the seizure phenotype.

The mechanism by which the *KCNH1* p.Val713Glu mutation leads to hyperexcitability still remains to be elucidated. Here, we utilized bioinformatical tools to predict the 3D structures of the wild-type and mutated protein. According to the prediction, the point mutation induced a conformational change at the C-terminus. Moreover, the mutated residue is located within the residues responsible for calmodulin binding (between amino acid positions 673 and 770). Under physiological conditions, binding of Ca^2+^/calmodulin inhibits channel activity [14,15,16]. Taken together, our analyses suggest that the p.Val713Glu mutation has an impact on the structure and function of the *KCNH1* protein, maybe by increasing potassium conductance. However, further functional studies will be required to improve our understanding of how the mutation contributes to the epilepsy phenotype.

## Figures and Tables

**Table 1:** Overview of novel *KCNH1* mutations detected in this study.

Patient	*KCNH1* Mutation	Inheritance	Phenotype
Case 1	p.Lys199Arg	de novo	DEE
Case 2	p.Arg535*	germline (pi)	GGE+
Case 3	p.Val713Glu	germline (mi)	GGE
Case 4	p.Val713Glu	somatic	FCD IIb

pi—paternal inherited; mi—maternal inherited; DEE—developmental and epileptic encephalopathy; GGE—genetic generalized epilepsy; GGE+—genetic generalized epilepsy with intellectual disability; FCD IIb—focal cortical dysplasia type IIb.

## Data Availability

No primary datasets were produced in this study. Data sharing is not applicable to this article.

## References

[1] Kortüm F., Caputo V., Bauer C.K., Stella L., Ciolfi A., Alawi M., Bocchinfuso G., Flex E., Paolacci S., Dentici M.L. (2015). Mutations in KCNH1 and ATP6V1B2 cause Zimmermann-Laband syndrome. Nat. Genet..

[2] Simons C., Rash L.D., Crawford J., Ma L., Cristofori-Armstrong B., Miller D., Ru K., Baillie G.J., Alanay Y., Jacquinet A. (2014). Mutations in the voltage-gated potassium channel gene KCNH1 cause Temple-Baraitser syndrome and epilepsy. Nat. Genet..

[3] Mastrangelo M., Scheffer I.E., Bramswig N.C., Nair L.D.V., Myers C.T., Dentici M.L., Korenke G.C., Schoch K., Campeau P.M., White S.M. (2016). Epilepsy in KCNH1-related syndromes. Epileptic Disord..

[4] Mégarbané A., Al-Ali R., Choucair N., Lek M., Wang E., Ladjimi M., Rose C.M., Hobeika R., Macary Y., Temanni R. (2016). Temple-Baraitser Syndrome and Zimmermann-Laband Syndrome: One clinical entity?. BMC Med. Genet..

[5] Miller S.A., Dykes D.D., Polesky H.F. (1988). A simple salting out procedure for extracting DNA from human nucleated cells. Nucleic Acids Res..

[6] Kudin A.P., Baron G., Zsurka G., Hampel K.G., Elger C.E., Grote A., Weber Y.G., Lerche H., Thiele H., Nürnberg P. (2017). Homozygous mutation in TXNRD1 is associated with genetic generalized epilepsy. Free. Radic. Biol. Med..

[7] Varbank. https://varbank.ccg.uni-koeln.de/varbank2/.

[8] Yang J., Anishchenko I., Park H., Peng Z., Ovchinnikov S., Baker D. (2020). Improved protein structure prediction using predicted interresidue orientations. Proc. Natl. Acad. Sci. USA.

[9] Laskowski R.A., MacArthur M.W., Thornton J.M. (2012). PROCHECK: Validation of protein-structure coordinates. International Tables for Crystallography.

[10] von Voss H., Schulz J., Borggraefe I., Kutsche K., Wilke C., Peraud A., Fellinger J., Marschall C., Heinrich U., Klein H. (2020). De novo variant in the KCNH1 gene associated with therapy refractory epilepsy. Paediatrische Praxis.

[11] Baldassari S., Ribierre T., Marsan E., Adle-Biassette H., Ferrand-Sorbets S., Bulteau C., Dorison N., Fohlen M., Polivka M., Weckhuysen S. (2019). Dissecting the genetic basis of focal cortical dysplasia: A large cohort study. Acta Neuropathol..

[12] Blümcke I., Sarnat H.B. (2016). Somatic mutations rather than viral infection classify focal cortical dysplasia type II as mTORopathy. Curr. Opin. Neurol..

[13] GnomAD. https://gnomad.broadinstitute.org/gene/ENSG00000143473?dataset=gnomad_r2_1.

[14] Alves J.T.G., Stühmer W. (2010). Calmodulin Interaction with hEAG1 Visualized by FRET Microscopy. PLoS ONE.

[15] Schönherr R., Löber K., Heinemann S.H. (2000). Inhibition of human ether a go-go potassium channels by Ca^2+^/calmodulin. EMBO J..

[16] Ziechner U., Schönherr R., Born A.-K., Gavrilova-Ruch O., Glaser R.W., Malešević M., Küllertz G., Heinemann S.H. (2006). Inhibition of human ether a go-go potassium channels by Ca^2+^/calmodulin binding to the cytosolic N- and C-termini. FEBS J..

